# A short-term in vivo model for giant cell tumor of bone

**DOI:** 10.1186/1471-2407-11-241

**Published:** 2011-06-13

**Authors:** Maurice Balke, Anna Neumann, Károly Szuhai, Konstantin Agelopoulos, Christian August, Georg Gosheger, Pancras CW Hogendoorn, Nick Athanasou, Horst Buerger, Martin Hagedorn

**Affiliations:** 1 Department of Trauma and Orthopedic Surgery, University of Witten-Herdecke, Cologne-Merheim Medical Center, Ostmerheimer Str., 200, 51109 Cologne, Germany; 2 Department of Orthopedic Surgery, University of Muenster, Albert-Schweitzer-Str. 33, 48149 Muenster, Germany; 3 Gerhard-Domagk-Institute of Pathology, University of Muenster, Domagkstr. 17, 48149 Muenster, Germany; 4 Department of Pathology, Leiden University Medical Center, Albinusdreef 2, 2300 RC, Leiden, The Netherlands; 5 Department of Molecular Cell Biology, Leiden University Medical Center, Einthovenweg 20, 2300 RC, Leiden, The Netherlands; 6 Department of Medicine, Hematology and Oncology, University of Muenster Domagkstrasse 3, 48149 Muenster, Germany; 7 Institute of Pathology, Klinikum Hanau GmbH, Leimenstr. 20, 63450 Hanau am Main, Germany; 8 Department of Pathology, Nuffield Orthopaedic Centre, University of Oxford, Oxford OX3 7LD, UK; 9 Institute of Pathology, Husener Str. 46 a 33098 Paderborn, Germany; 10 INSERM U1029, Avenue des Facultés, Bâtiment B2, 33405 Talence cedex, France; 11 University Bordeaux 1, Avenue des Facultés, Bâtiment B2, 33405 Talence cedex, France

## Abstract

**Background:**

Because of the lack of suitable *in vivo *models of giant cell tumor of bone (GCT), little is known about its underlying fundamental pro-tumoral events, such as tumor growth, invasion, angiogenesis and metastasis. There is no existing cell line that contains all the cell and tissue tumor components of GCT and thus *in vitro *testing of anti-tumor agents on GCT is not possible. In this study we have characterized a new method of growing a GCT tumor on a chick chorio-allantoic membrane (CAM) for this purpose.

**Methods:**

Fresh tumor tissue was obtained from 10 patients and homogenized. The suspension was grafted onto the CAM at day 10 of development. The growth process was monitored by daily observation and photo documentation using *in vivo *biomicroscopy. After 6 days, samples were fixed and further analyzed using standard histology (hematoxylin and eosin stains), Ki67 staining and fluorescence *in situ *hybridization (FISH).

**Results:**

The suspension of all 10 patients formed solid tumors when grafted on the CAM. *In vivo *microscopy and standard histology revealed a rich vascularization of the tumors. The tumors were composed of the typical components of GCT, including (CD51+/CD68+) multinucleated giant cells whichwere generally less numerous and contained fewer nuclei than in the original tumors. Ki67 staining revealed a very low proliferation rate. The FISH demonstrated that the tumors were composed of human cells interspersed with chick-derived capillaries.

**Conclusions:**

A reliable protocol for grafting of human GCT onto the chick chorio-allantoic membrane is established. This is the first *in vivo *model for giant cell tumors of bone which opens new perspectives to study this disease and to test new therapeutical agents.

## Background

Giant cell tumor of bone (GCT) is an aggressive skeletal lesion typically located in the epiphyseal end of a long bone [1-3]. The tumor predominantly occurs in the third and fourth decade of life with a slight predilection for females [3-8].

GCT is characterized by locally aggressive growth usually leading to extensive bone destruction [9]. The biological behavior of the tumor is, however unpredictable, and attempts to histologically grade the tumors have failed [10-12]. At the genomic level however recurrent cases are characterized by random individual cell aneusomy, while malignant cases show abnormalities at array CGH level [13].

GCT is characterized by the presence of numerous Cathepsin-K producing, CD33 +, CD14 - multinucleated osteoclast-like giant cells and plump spindle-shaped stromal cells that represent the main proliferating cell population [14-17]. The spindle-shaped mononuclear cells are believed to represent the neoplastic population and are characterized at the cytogenetic level by telomeric associations and a peculiar telomere-protecting capping mechanism [18]. Areas of regressive change such as necrosis or fibrosis as well as extensive hemorrhage are frequently present.

The treatment of choice is intralesional curettage and bone cement packing leading to a local recurrence rate of 10 to 40% [1,19,20]; treatment options are limited and recurrence rates are higher when GCT arises at a surgical inaccessible location (e.g. spine and sacrum). In addition, some GCT may rarely arise at multiple sites or undergo sarcomatous transformation. In about 2% of cases, patients develop lung metastases, which are thought to represent benign pulmonary implants that arise following vascular invasion [21-25].

The underlying pathobiology of GCT growth and development of these complications is unknown. There is no successful adjuvant treatment option, although there are reports of a limited effect on tumor growth following treatment with bisphosphonates [26,27] and anti-RANKL antibodies [28], agents that inhibit the formation and activity of the osteoclastic giant cells in the tumor.

Thus far, attempts to grow GCT in animal models as well as to derive suitable cell lines from primary tumors have failed. This has limited the study of pathobiology of GCT and the development of specific anti-GCT agents. To address this problem we have examined whether it is possible to establish the growth of GCT short-term *in vivo *in a chick chorio-allantoic membrane (CAM) assay.

The CAM is characterized by an extremely dense vascular network with large vessels situated within the somatic mesoderm and capillaries located within or directly under the splanchnic mesoderm. This double-layer membrane develops by fusion of the chorion with the allantoic vesicle on embryonic day 4 - 5 [29]. Until hatching the CAM physiologically absorbs calcium from the shell, stores waste products and serves as a respiratory organ [30].

The CAM assay has been utilized as a model system for more than a century to demonstrate development of embryonic blood vessels, and to provide a host for the grafting of bacteria, viruses and embryonic tissue. In the last 25 years, the CAM assay has become established as a model for angiogenesis research; this has been used to provide highly reproducible models for aggressive and malignant tumors including glioblastoma and pancreatic adenocarcinoma [31,32].

The use of the CAM assay in bone tumor research has only been sporadically reported. We recently published the successful establishment of human osteosarcoma cell lines on a CAM assay and provided evidence that the MNNG-HOS cell line reproduces the key features of human osteosarcoma growth when grafted on the CAM [33]. This relatively simple experimental approach enables tumor growth and vascularization to be easily studied and permits the growth of tumors to be studied in an inexpensive way.

In this report, we present the results of successful establishment of human GCT in a CAM assay with emphasis on the morphological characteristics of the grafted tumors.

## Methods

### Patients

The patients included in this study had typical, histologically confirmed cases of giant cell tumors of bone (GCT). The mean age of the five male and five female patients was 29.8 years; eight of ten were localized in the extremities, one in the spine and one in the pelvis. Four were recurrent cases (see Additional file 1). All patients gave their written consent prior to tumor tissue isolation for research studies. All samples were handled in a coded fashion and the experiments were performed according to the local ethical guidelines.

### Giant cell suspension

Cell suspensions isolated from GCT tissue of 10 patients were used in the experiment. Tissue samples were minced and incubated at 37°C in RPMI with 5-10 ml DNAse (2200 KU/100 ml - Sigma-Aldrich, Germany; cat. no. DN-25-10MG) and 5-10 ml collagenase Type 2 (500 U/ml - PAA; Austria; cat. no. K21-240) for 3-8 hours. DNAse and collagenase solutions were mixed in equal parts. The homogenized tissue solution was centrifuged at 1200 rpm for 5 min and the cell pellet was subsequently washed twice with RPMI 1640 (PAA Austria; cat. no. E15-840) supplemented with 10% Foetal Bovine Serum FBS Gold (PAA Austria; cat. no. A15-649) and 1% penicilline/streptomycine (PAA Austria; cat. no. P11-010). This procedure was repeated four times.

### Freezing giant cell suspension

After the last washing step, the cell pellet was re-suspended in CryoMaxx S freezing medium (PAA, Austria cat. no. J05-013 - approximately 50 μl to 200 μl cells per ml freezing medium). One ml suspension was frozen per cryotube (Nunc; Germany; cat. no. 368632). Finished vials were frozen overnight at -70°C in a freezing container (NALGENE^® ^Labware, Hereford, United Kingdom Cat. No. 5100-0001) and stored in liquid nitrogen.

### Thawing giant cell suspension

Cell culture medium RPMI 1640 (PAA Austria; cat. no. E15-840) supplemented with 10% Foetal Bovine Serum FBS Gold (PAA Austria; cat. no. A15-649) and 1% penicilline/streptomycine (PAA Austria; cat. no. P11-010) was preheated at 37°C and 50 ml were propounded in a conical centrifuge tube. The frozen vial of giant cells was thawed in a 37°C water bath to that point that it was possible to decant the cells into the RPMI (a rest of ice in the tube is necessary). The cells were decanted into the RPMI medium and centrifuged at 1200 rpm for 5 min. The resultant cell pellet was subsequently washed with RPMI 1640 four times. The yield of isolated cells was re-suspended and seeded on the day 10 CAM (20 μl each).

### The chick chorio-allantoic membrane assay

Fertilized white leghorn chicken eggs (Valo-SPF eggs, Lohmann Tierzucht GmbH, Cuxhaven, Germany) were incubated at a humidity of 70% and 37°C. At embryonic day 3, 2 - 3 ml of albumen were removed with a syringe, thus allowing detachment of the embryo and a small window was cut into the eggshell. After verification of normal development of the embryo the window was sealed with tape. After 10 days of incubation small plastic rings made out of Thermanox™ cover discs were placed on the CAM. After gentle laceration of the CAM surface 20 μl of re-suspended tumor suspension were deposited into the rings. For the controls only 20 μl of RPMI was used.

Until day 16 CAMs were examined and photographed *in ovo *with a digital camera (Olympus E330) attached to a stereomicroscope. All embryos that died before day 16 were excluded from further analyses. Tumor volumes were estimated by the following formula: V = 4/3*p*r^3 ^(r = 1/2 * square root of diameter 1 * diameter 2) [31].

For further information of the technique of the CAM assay see instructional videos in the 'additional files' section (Additional files 2, 3, 4, 5, 6 and 7).

### Histology and Immunohistochemistry

At embryonic day 16, (6 days of tumor growth), tumors were fixed *in vivo *using 4% paraformaldehyde for 20 min. Tumors were removed and transferred into culture dishes and samples were observed and photographed. Relevant samples were embedded in paraffin and cut into 10 μm sections. Tissue sections were stained with hematoxylin-eosin and by immunohistochemistry using an indirect immunoperoxidase technique, with mouse monoclonal antibodies MIB-1, and KPI (both obtained from DAKO-UK) and NCL-CD14 and NCL-CD51 (Novocastra, UK) directed against the proliferation marker Ki67, the macrophage/osteoclast marker CD68, the monocyte/macrophage marker CD14, and the osteoclast marker CD51 (vitronectin receptor) respectively. Results were analyzed by standard light microscopy (Leica DM2500 with Leica EC3 camera).

### Interphase fluorescence in situ hybridization (FISH)

for the positive identification of cells with human origin we performed an interphase FISH using human haploid repeat sequence containing probe sets [34]. These alpha-satellite probes specifically recognize (peri)centromeric sequences of human chromosomes. Based on the size and specificity of these alpha-satellite probes we selected human chromosome 1 (PUC 1.77) and 15 (D15Z1) [35].

Interphase FISH was performed according to previously described protocols on formalin-fixed paraffin-embedded tissue slides [36]. Chromosome 1 (detected by FITC, green) and chromosome 15 (detected by Cy3, red) specific alpha satellite probes were labeled by using standard nick translation procedure, hybridized and analyzed as previously described [37]. All slides were embedded in Citifluor anti-fading solution containing DAPI for visualization of DNA of the interphase nuclei.

## Results

### In vivo observation

All of the ten GCT samples were able to form solid vascularized tumors when grafted to the CAM (Additional file 1, Figure 1 and 2). No significant differences in the growth rate were observed according to the primary lesion. The percentage of tumors after 6 days of growth in living embryos was 86.9% (60 of 69). The overall death rate after grafting of the tumor tissue was 55% (69 of 125) and was significantly higher (P = 0.001, Fisher's exact test - Figure 3) than the death rate of the controls, which was 19% (5 of 26).

**Figure 1:**
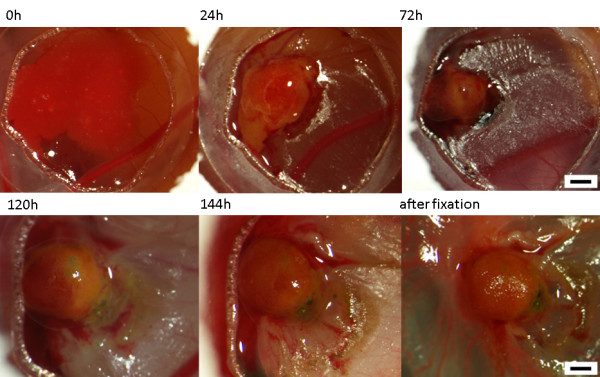
**In vivo observations of tumor growth**. The tumor solute is seeded into the plastic ring on the CAM (0h). After 24 h a solid tumor develops which gets further vascularized (24 to 144 h). The typical red/yellow-brownish color as well as areas of haemorrhage are visible. Upper row magnification 10 ×, scale bar 1 mm; lower row magnification 20×, scale bar 500 μm.

**Figure 2:**
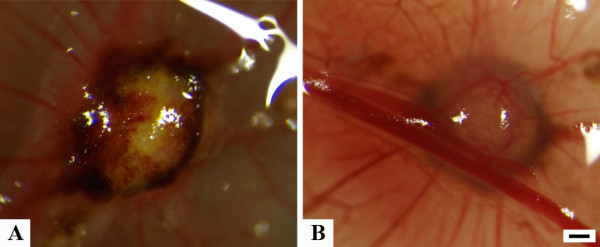
**Photographs of day 6 tumor**. Another example of a GCT grown on the CAM. Note the typical yellow-brownish color in **A **and the strong vascularization of the tumor when the CAM is turned upside down after fixation in **B**. Magnification 40 ×, scale bar 250 μm.

**Figure 3:**
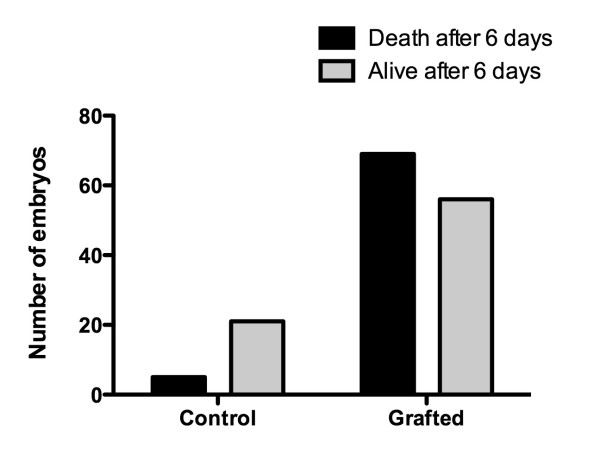
**Survival of embryos after tumor grafting**. The overall death rate after grafting of the tumor tissue is siginficantly higher than the death rate of the controls. P = 0.001, Fisher's exact test.

24h after grafting of the suspension, a solid tumor became apparent which then progressively further vascularized without significantly increasing in size (Figure 1). With the typical yellow-brownish color and the strong vascularization the tumors resembled the macroscopical aspect of GCT during surgery (Figure 2). The overall mean estimated tumor volume was 12.3 mm^3 ^(4.3 - 35.6 mm^3^, Additional file 1).

### Histological and Immunohistochemical findings

**Figure 4:**
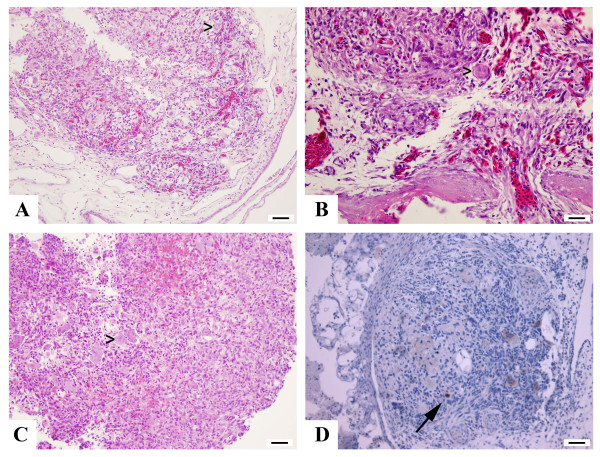
**Histology and Ki67 staining**. Hematoxylin-eosin stain of a day 6 tumor (**A **and **B**) shows all cell components of a GCT and closely resembles original tumor (**C**). Note the fewer giant cells (**arrowheads**) in A and B containing fewer nuclei compared to original tumor in **C**. Note the very low proliferation activity (**arrow**) in the nuclear staining of MIB-1 in **D**. Magnification in A+C 20 × (scale bar 50 μm), B: 40 × (scale bar 25 μm), D: 10 × (scale bar 100 μm).

**Figure 5:**
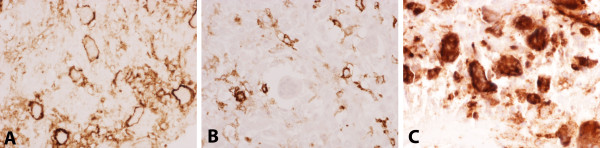
**Immunophenotypic profile of giant cells**. Typical immunophenotypic profile of osteoclasts, being CD51 + (**A**) and CD14 - (**B**). Giant cells reacted for CD68 (**C**), which is expressed by both macrophages and osteoclasts. Magnification 400 ×.

The tumor samples cultured on the CAM contained both (osteoclast-like) giant cell and mononuclear components of GCT (Figure 4). The giant cells reacted for CD68, which is expressed by both macrophages and osteoclasts, and exhibited the typical immunophenotypic profile of osteoclasts, being CD14- and CD51+ (Figure 5); giant cells in GCT exhibit a similar antigenic phenotype [38,39]. The mononuclear component contained cells expressing CD68, CD14 and CD51. Giant cells were numerous and widely scattered throughout the original tumors but fewer were noted in tumors cultured on the CAM. Tumor giant cells frequently contained more than five nuclei in the original tumors but were smaller and contained fewer nuclei in the cultured samples. The tumors appear to grow on the membrane rather than invade it, producing an implant-like rather than infiltrative growth pattern. Vessels were recruited from the CAM to vascularize the tumor. Ki-67 revealed a very low proliferating fraction (less than 1%) of cells. The tumors contained a background chronic inflammatory cell infiltrate including lymphocytes and plasma cells.

### Interphase fluorescence in situ hybridization (FISH)

**Figure 6:**
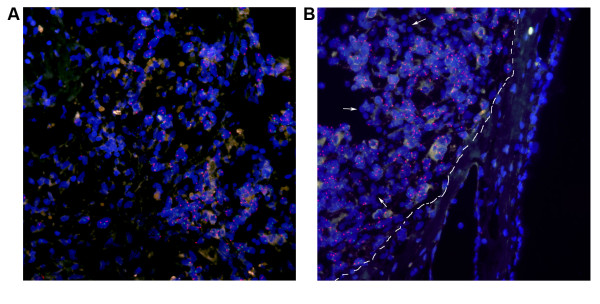
**Fluorescence in situ hybridization**. Interphase FISH overview using probes specific to human chromosome 1 (green) and chromosome 15 (red) alpha-satellite sequences. Sections were counterstained by DAPI (blue) showing nuclei of both human and chicken cells. **A**: FISH signals were detected in human cells only, chicken erythrocytes showed no signals (indicated by white arrows). **B**: Human cells are well demarcated from CAM cells (dashed white line) and attracted numerous blood vessels and erythrocytes (white arrows).

For the discrimination between the human and chicken cells, we performed interphase FISH using human alpha-satellite probes specific to the heterochromatic region of chromosome 1q12 and the (peri)centromeric region of chromosomes 15. The two color labeling of these two probes allows the identification of human cells with FISH signals while chicken cells would be stained with DAPI only. The use of a similar approach to discriminate between human and mouse cells have been shown by us earlier [34]. Despite the very strong auto-fluorescence coming from extracellular matrix material of the CAM, a clear recognition of the FISH positive human cells were possible (Figure 6). FISH image using two human centromeric probes (red, green) showed that there was no cross reactivity between human and chicken centromeres. There was no signal in the CAM nor in the remaining chicken erythrocytes in the tumor nor in the vascular endothelium (Figure 6B). The giant cells were positive for FISH indicating that they were of human origin.

## Discussion

GCT is a locally aggressive primary tumor of bone that commonly recurs and in some cases can be life threatening [40]. Control of tumor growth and intervention to reduce complications such as the development of pulmonary metastases has hitherto not been possible [1,41]. The major problem in GCT research is the lack of animal models in which to study GCT growth and pathobiology. Although mononuclear stromal cells can be cultured from GCT, other significant cellular components, notably macrophages and giant cells, do not remain in culture after passaging [42,43]. Due to these difficulties, very little is known on the pathobiology of this particular tumor.

The CAM assay is an established *in vivo *model to study angiogenesis [44]. It is characterized by several advantages such as easy accessibility and relatively simple and cost effective experimental approach. Despite its natural immunodeficient environment (for review see [45]) the CAM assay is still rarely used for tumor grafting. There are reports about the use of the CAM assay as a reliable model to study tumors such as glioblastoma [31], prostatic cancer [46], and melanoma [47], but compared to murine models of tumor growth these are relatively rare. Reports of the use of the CAM assay for establishment of human bone and soft tissue tumors are largely anecdotal [48,49].

Recently, we established the CAM assay for human osteosarcoma cell lines and were able to show that the MNNG-HOS, U2OS and SAOS cell lines consistently developed vascularized tumors that simulated key features of human osteosarcoma growth such as angiogenesis, necrosis and hemorrhage [33]. Compared to tumor formation with osteosarcoma cell lines, where the number of successful implanted tumors was around 50%, the number of tumors which resulted from suspensions of GCTs seeded on the CAM was nearly 90% (60 of 69). However, the overall embryonic mortality rate after tumor grafting was 55% (69 of 125); this was higher than in the previous osteosarcoma study where there was an embryonic mortality rate of 38% (58 of 152) [33]. The underlying reasons for this difference remain speculative, but it is possible that the mortality rate may correlate with tumor (graft) and host interactions; death might be caused by tumor cell dissemination directly or via secretion of specific factors causing blood coagulation. Metabolic stress such as hypoxia and/or serum deprivation [50], may occur in the tumors grafted to the CAM and might induce tumor cells to secrete such molecules.

One theory of GCT lung metastasis formation is that tumor cells invade blood vessels and are disseminated via the blood stream, finally implanting in the lungs. Following implantation, GCTs become established by inducing angiogenesis and further vascularization. The cultured GCTs in our model appeared to grow on the CAM and showed some morphological resemblance to metastatic GCT lung nodules. Although the tumors which grew on the CAM contained all the cellular components of GCT, including stromal cells, macrophages and multinucleated osteoclast-like giant cells, the latter were generally less numerous than in the original tumor, a morphological finding which has been noted in GCT lung nodules [51]. Areas of necrosis and haemorrhage as well as extensive vascularization were present in the tumors grown on the CAM, all features which are typical for GCT. The Ki67 staining demonstrated a very low proliferation rate, in contrast to the original tumors. This might be due to the short time span (only 6 days) of tumor growth. Thus the model would appear to simulate the early phase of tumor seeding, one of the initial steps in the development of a metastasis or local recurrence.

The FISH analysis provided evidence that the experimental GCTs on the CAM are hybrid tumors composed of human graft and chicken host (vasculature) cells. Therefore the model might allow further studies clarifying these crucial steps of angiogenesis and tumor invasion and metastasis which are promising targets for new drugs against solid tumors [52,53]. As an example it might be possible to develop new antitumor drugs by simultaneous measuring of gene expression using Affymetrix chicken GeneChips and human GeneChips in the tumor cells as well as in newly formed blood vessels, as has been shown for pancreatic adenocarcinoma [32]. This model represents an excellent alternative to the commonly used animal models. It is cost effective and fulfills the recommendations of an ethically appreciable use of live animals in cancer research [54]. The fact that tumors were grown from a frozen cell suspension will also favor exchange of material between different centres, an important point given the rarity of GCT.

The typical GCT can usually be locally controlled by intralesional curettage or surgical removal. The treatment options for complicated cases, such as GCT with pulmonary metastases or GCT arising in a surgically inaccessible site, are limited. To date only a few promising therapies have been developed for adjuvant use in these cases. The few publications that exist on systemic treatment of GCT have focused on inhibition of osteoclastic bone resorption with bisphosphonates or disruption of the RANK/RANKL pathway of osteoclast formation with specific antibodies such as denosumab [28,55]. The limitation of this treatment is that only osteoclasts are inhibited, whereas the proliferating neoplastic stromal cells are mainly unaffected. Thus these treatments might only have a short term effect. Use of the CAM assay should permit the effect of therapeutic agents on both stromal and giant cell components of GCT to be studied in greater detail; this model should also facilitate further molecular characterization of the cellular components of this rare tumor.

## Conclusions

A reliable protocol for grafting of human GCT onto the chick chorio-allantoic membrane is established. This is the first *in vivo *model for giant cell tumors of bone which opens new perspectives to study this disease and to test new therapeutical agents.

## Competing interests

The authors declare that they have no competing interests.

## Authors' contributions

MB, AN, NA, KS, and MH designed experiments and wrote the manuscript. MB, AN, KA, KS, KA, and HB conducted the experiments. MB, and GG collected the patient data and performed the surgeries. CA, NA and HB performed the histology and immunohistochemistry. KS, and PH performed the FISH analysis. MB and MH developed the ideas, NA and PH corrected and edited the manuscript. All authors have read and approved the final manuscript.

## Pre-publication history

The pre-publication history for this paper can be accessed here:

http://www.biomedcentral.com/1471-2407/11/241/prepub

## Supplementary Material

Additional file 1 **Table 1**. Information on patients, anatomical localization of tumor, mortality/growth rate and tumor size. M = male, F = Female, prox = proximal, dist = distal, R = Recurrence, V mm^3 ^= mean tumor volume calculated by V = 4/3*p*r^3 ^(r = 1/2 * square root of diameter 1 * diameter 2), SD = standard deviation. Click here for file

Additional file 2 **Opening of the eggs**. Video showing the process of opening of the egg. Click here for file

Additional file 3 **Preparation of plastic rings**. Video demonstrating the preparation of the plastic rings. Click here for file

Additional file 4 **Placement of the plastic ring on the CAM**. Video showing the placement of the plastic ring on the CAM. Click here for file

Additional file 5 **Gentle laceration of the CAM surface**. Video showing the process of gentle laceration of the CAM surface. Click here for file

Additional file 6 **Grafting of the tumor cells**. Video demonstrating the technique of tumor cell grafting. Click here for file

Additional file 7 **Fixation and further processing of the tumor tissue**. Video demonstration the method of fixation and preparation of the tissue for further processing. Click here for file
