# Supplementary material for: A short-term in vivo model for giant cell tumor of bone
Source: BMC Cancer. 2011 Jun 13;11:241. doi: 10.1186/1471-2407-11-241 (PMC3125284; doi:10.1186/1471-2407-11-241)
Supplement: Additional file 1 — Table 1. Information on patients, anatomical localization of tumor, mortality/growth rate and tumor size. M = male, F = Female, prox = proximal, dist = distal, R = Recurrence, V mm3 = mean tumor volume calculated by V = 4/3*p*r3 (r = 1/2 * square root of diameter 1 * diameter 2), SD = standard deviation. [file 1471-2407-11-241-S1.DOC]

| **Patient** | **localization** | **n d0** | **n alive d6** | **n tumor d6** | **V mm³** | **SD** |
| --- | --- | --- | --- | --- | --- | --- |
| **1) 37, M** | Tibia prox, R1 | 12 | 6 | 6 | 9.7 | 4.98 |
| **2) 24, M** | Fibula prox, R1 | 10 | 5 | 5 | 35.6 | 4.44 |
| **3) 27, F** | Thorac. spine | 4 | 1 | 1 | 4.3 | 0.00 |
| **4) 31, M** | Femur dist, R1 | 19 | 10 | 10 | 13.5 | 13.17 |
| **5) 20, F** | Femur prox | 13 | 8 | 6 | 4.3 | 2.79 |
| **6) 18, F** | Tibia prox | 22 | 10 | 8 | 13.6 | 14.65 |
| **7) 20, F** | Humerus prox | 8 | 2 | 2 | 1.5 | 0.00 |
| **8) 70, F** | Radius dist, R2 | 10 | 9 | 8 | 12.9 | 7.96 |
| **9) 19, M** | Femur dist | 14 | 8 | 6 | 16.5 | 16.33 |
| **10) 32, M** | Pelvis | 13 | 10 | 8 | 11.4 | 10.81 |
